# Supplementary material for: Calculating forest species diversity with information-theory based indices using sentinel-2A sensor’s of Mahavir Swami Wildlife Sanctuary
Source: PLoS One. 2022 May 18;17(5):e0268018. doi: 10.1371/journal.pone.0268018 (PMC9116646; doi:10.1371/journal.pone.0268018)
Supplement: Attachment — Submitted filename: Response to reviewers.docx [file pone.0268018.s002.docx]

**Reviewer 1**

**Comments**

The title should be comprehensive and cover all steps of investigation.

**Justification:** Title has been revised according to cover all steps of investigation.

Calculating forest species diversity with information-theory based indices using sentinel-2A sensor’s of mahavir swami wildlife sanctuary.

**Comments**
Abstract should be facile and understandable to readers with technical words.

**Justification:** Abstract has been revised according to understandable to readers with technical words.

Tropical forest serves as an important pivotal role in terrestrial biological diversity. The present study makes an attempt to identify the concentration of species among tree diversity in MSWS. Four important ecological indicator indices namely Shannon-Weiner index (H’), Simpson’s diversity (D), Margalef index (SR) and Pielou's (J) indices were make the most for species diversity measurement. The research outcomes revealed that Shannon-Weiner diversity index (H/) was found to be the best index for assessing species richness while Simpson's diversity (D) index was more suited for determining species diversity. The Shannon-Weiner index value calculated for different transects not only represent the species richness but also the species evenness in each transect. The potential application of forest diversity can be used a mechanism for forest management. The methodology will retrofit better policy implementation for maintaining the health of forest species in mahavir swami wildlife sanctuary and can be applied on other reserve forest of socio-ecological significance.

**Comments**
Most recent work should be cited in introduction.

**Justification:** Recent RoL has been cited in introduction part.

a. Wang, R.; Gamon, J.A. Remote sensing of terrestrial plant biodiversity. Remote Sens. Environ. 2019, 231, 111218.
b. Turner, W.; Rondinini, C.; Pettorelli, N.; Mora, B.; Leidner, A.K.; Szantoi, Z.; Buchanan, G.; Dech, S.; Dwyer, J.; Herold, M.; et al. Free and open-access satellite data are key to biodiversity conservation. Biol. Conserv. 2015, 182, 173–176
c. Alleaume, S.; Dusseux, P.; Thierion, V.; Commagnac, L.; Laventure, S.; Lang, M.; Féret, J.-B.; Hubert-Moy, L.; Luque, S. A generic remote sensing approach to derive operational essential biodiversity variables (EBVs) for conservation planning. Methods Ecol. Evol. 2018, 9, 1822–1836.
d. Rocchini, D.; Boyd, D.S.; Féret, J.-B.; Foody, G.M.; He, K.S.; Lausch, A.; Nagendra, H.; Wegmann, M.; Pettorelli, N. Satellite remote sensing to monitor species diversity: Potential and pitfalls. Remote Sens. Ecol. Conserv. 2016, 2, 25–36.

**Comments**
Line 150-151: show the research gap of Calculating Forest Diversity in study area that leads the author to conduct the present research.

**Justification:** We completely agree and have updated the sentence accordingly.

The present study makes an attempt to calculate species diversity indices and structural forms of the tropical forest.

**Comments**
Material section similar contents if merged together

**Justification:** We completely agree and have updated the section accordingly

Section 2.2 and 2.3 has been merged.

Sentinel-2A data based biodiversity extent

**Comments**
Blurry figures so, resolve this problem and also revise the figure captions with detailed illustrations

**Justification:** We completely agreed all figures have been imported up to 300 DPI. May be in PDF version it show some low resolution.

**Comments**
Forest Diversity is key indicator of study should be properly defined in results.

**Justification:** The manuscript was substantially revised throughout in result part.

Tropical forest acts as munificence for the life forms living in the tropics, by providing habitat conditions and natural resources. Analytical scrutiny of the forest stand is provided by the structural diversity of forest and can be sub divided into three categories; tree species diversity, tree dimension diversity and tree position diversity. Forest is the key indicator of this research.

**Reviewer: 2**
**Comments**
The main method used in this study are data collection and indices calculation.

**Justification:** We completely agree and have updated the methodology.

**Comments**
What are the innovations? I cannot see.

**Justification:** I have removed this world from the manuscript.

**Comments**
A comprehensive literature is required for identifying the research gaps and highlight the necessity for carrying out this study.

**Justification:** Recent RoL has been cited in introduction part.

e. Wang, R.; Gamon, J.A. Remote sensing of terrestrial plant biodiversity. Remote Sens. Environ. 2019, 231, 111218.
f. Turner, W.; Rondinini, C.; Pettorelli, N.; Mora, B.; Leidner, A.K.; Szantoi, Z.; Buchanan, G.; Dech, S.; Dwyer, J.; Herold, M.; et al. Free and open-access satellite data are key to biodiversity conservation. Biol. Conserv. 2015, 182, 173–176
g. Alleaume, S.; Dusseux, P.; Thierion, V.; Commagnac, L.; Laventure, S.; Lang, M.; Féret, J.-B.; Hubert-Moy, L.; Luque, S. A generic remote sensing approach to derive operational essential biodiversity variables (EBVs) for conservation planning. Methods Ecol. Evol. 2018, 9, 1822–1836.
h. Rocchini, D.; Boyd, D.S.; Féret, J.-B.; Foody, G.M.; He, K.S.; Lausch, A.; Nagendra, H.; Wegmann, M.; Pettorelli, N. Satellite remote sensing to monitor species diversity: Potential and pitfalls. Remote Sens. Ecol. Conserv. 2016, 2, 25–36
